# Design, Fabrication, and Validation of a Petri Dish-Compatible PDMS Bioreactor for the Tensile Stimulation and Characterization of Microtissues

**DOI:** 10.3390/mi11100892

**Published:** 2020-09-26

**Authors:** Soliman Alhudaithy, Sama Abdulmalik, Sangamesh G. Kumbar, Kazunori Hoshino

**Affiliations:** 1 Department of Biomedical Engineering, University of Connecticut, Storrs, CT 06269, USA; soliman.alhudaithy@uconn.edu (S.A.); sama.abdulmalik@uconn.edu (S.A.); kumbar@uchc.edu (S.K.); 2 Department of Biomedical Technology, King Saud University, Riyadh 12372, Saudi Arabia; 3 Department of Orthopedic Surgery, University of Connecticut Health, Farmington, CT 06030, USA; 4 Department of Materials Science and Engineering, University of Connecticut, Storrs, CT 06269, USA

**Keywords:** polydimethylsiloxane (PDMS), soft-lithography, MEMS, micromanipulation, actuator, bioreactor, nanofibers, mechanical stimulation, cell-material interaction, biomechanics

## Abstract

In this paper, we report on a novel biocompatible micromechanical bioreactor (actuator and sensor) designed for the in situ manipulation and characterization of live microtissues. The purpose of this study was to develop and validate an application-targeted sterile bioreactor that is accessible, inexpensive, adjustable, and easily fabricated. Our method relies on a simple polydimethylsiloxane (PDMS) molding technique for fabrication and is compatible with commonly-used laboratory equipment and materials. Our unique design includes a flexible thin membrane that allows for the transfer of an external actuation into the PDMS beam-based actuator and sensor placed inside a conventional 35 mm cell culture Petri dish. Through computational analysis followed by experimental testing, we demonstrated its functionality, accuracy, sensitivity, and tunable operating range. Through time-course testing, the actuator delivered strains of over 20% to biodegradable electrospun poly (D, L-lactide-co-glycolide) (PLGA) 85:15 non-aligned nanofibers (~91 µm thick). At the same time, the sensor was able to characterize time-course changes in Young’s modulus (down to 10–150 kPa), induced by an application of isopropyl alcohol (IPA). Furthermore, the actuator delivered strains of up to 4% to PDMS monolayers (~30 µm thick), simultaneously characterizing their elastic modulus up to ~2.2 MPa. The platform repeatedly applied dynamic (0.23 Hz) tensile stimuli to live Human Dermal Fibroblast (HDF) cells for 12 hours (h) and recorded the cellular reorientation towards two angle regimes, with averages of −58.85° and +56.02°. The device biocompatibility with live cells was demonstrated for one week, with no signs of cytotoxicity. We can conclude that our PDMS bioreactor is advantageous for low-cost tissue/cell culture micromanipulation studies involving mechanical actuation and characterization. Our device eliminates the need for an expensive experimental setup for cell micromanipulation, increasing the ease of live-cell manipulation studies by providing an affordable way of conducting high-throughput experiments without the need to open the Petri dish, reducing manual handling, cross-contamination, supplies, and costs. The device design, material, and methods allow the user to define the operational range based on their targeted samples/application.

## 1. Introduction

Micromanipulation is an essential part of the study of cell and microtissue mechanical characteristics [1]. It is known that the mechanical properties are correlated with healthy or diseased statuses of cells and tissues [2,3,4,5]. The mechanical properties of substrates can influence the growth of cells cultured for regenerative tissue bioengineering applications [6,7,8]. For the study of bioengineered tissue reconstruction, the mimicry of an in vivo environment that provides relevant physiological stimuli to cells is vital [7]. The application of mechanical stimuli to microtissues on a cellular level is one fundamental approach.

Several effects of tensile stimulation have been reported, including enhanced matrix organization and proliferation on gelatin-matrix scaffolds seeded with human heart cells [9], an increased tissue mechanical strength in collagen with suspended skeletal muscle cells [10], and escalated elastin expression and tissue organization in polymeric scaffolds seeded with smooth muscle cells [11]. When stimulated, cells rearrange their cytoskeleton anchorage to the substrate based on its mechanical cues [12].

The cellular reorientation resulting from substrate tensile stretching has also been well-studied. Static and quasi-static stimuli tend to align cells parallel to the direction of stretching [13], while dynamic stimuli re-orient fibroblast cells to a well-defined perpendicular angle away from the direction of stretching [13,14]. Others have studied the effect of the strain amplitude and cyclic stretching frequency on fibroblast reorientation, where lower applied strain decreased the degree of perpendicular orientation [14,15], and the time required for reorientation increased [14,16]. Similarly, when the frequency of dynamic stimuli was decreased, the reorientation characteristic time increased with a power law. Frequencies between 0.0001 and 20 Hz and strain amplitudes from 1% to 15% have been tested, and thresholds have been established; with a lower frequency (0.1 Hz) and strain (2% strain), a period of time varying between 1 and 5 h based on the applied frequency and strain was sufficient for human dermal fibroblast (HDF) cell reorientation to occur.

Furthermore, frequencies above 1 Hz saturated the characteristic time [16], while an upper strain limit of 4.2 ± 0.4% was reported for fibroblasts’ presence [15]. To adapt the cellular thresholds, a focal adhesion method considering the mechanics of the substrate, cell stress fibers, and adhesion bonds has also been reported, where, beyond high frequency and strain thresholds, the stability of the adhesion cluster was disrupted [17]. All of these studies have investigated the cellular aspects of micromanipulations based on actuator stimuli.

The mechanical sensing and characterization of bioengineered tissues is another essential aspect. It is common to conduct end-point, non-sterile mechanical characterization after tissue growth in a separate bioreactor [18,19]. However, in situ mechanical characterization of live tissues growing in a bioreactor could provide valuable information, as it could help researchers to decide when the samples are ready and functional for particular applications based on their biological responsiveness and mechanical integrity over the mechanical conditioning time [6,20,21]. To conduct in situ, time-course tensile mechanical analysis, tissue manipulation must be conducted in a sterile environment. The bioreactor also has to fulfill a set of required functions and features [8]. Bioreactors designed for the above-discussed applications are required to provide critical functions, including the culturing of bio-samples in biocompatible chambers, the delivery of mechanical stimuli, the measurement of delicate forces, the circulation of media, the preservation of sterility [21,22,23], and preferably compatibility with laboratory standard incubators and imaging apparatus. Several commercially available tensile bioreactors offer these functions, including and not limited to, TC-3F by ©HypOxygen, MCJ1 by CellScale, BioTense Perfusion by ADMET Inc., ElectroForce^®^ BioDynamic^®^ by TA Instruments, and LigaGen™ by Bangalore Integrated System Solutions Ltd. However, such commercial setups may pose financial, technical, and scientific challenges [24]. Other laboratory-customized bioreactors, for example, are usually developed per application [14,16,20,25,26,27] and are not necessarily cost-effective or easy to fabricate, modify, tune, and scale-up without cost, access, and producibility concerns.

The purpose of this study was to develop a biocompatible micromechanical manipulation platform that is affordable, accessible, scalable, modifiable, and easily fabricated. The aim was to eliminate the need for experimental setups and fabrication resources that come with a price tag while delivering the bioreactor’s essential functions and compatibility with standard lab equipment. This paper demonstrates the design and validation of a uniaxial tensile actuator/sensor made of polydimethylsiloxane (PDMS) with a predictable and tunable operating range based on the targeted specimens/applications. The device is composed of two main components. The first component is the lid actuator, which replaces the regular lid of a cell culture Petri dish. It uniquely utilizes a flexible thin membrane that allows for the transfer of a linear actuation outside the Petri dish into an internal manipulation inside the Petri dish. The second component is the actuating/sensing stage, which fits inside a 35 mm Petri dish. Connected by a unique tunable link mechanism, the system was utilized as a time-course tensile bioreactor delivering mechanical micromanipulations while acquiring sample stiffness properties. Such a system will considerably increase the efficacy of tissue and cell manipulation targeted studies by providing an affordable way of testing many samples in parallel using available conventional lab equipment.

## 2. Methods

### 2.1. Design and Finite Element Method Analysis

We used Solidworks (Version 2019, Dassault Systèmes SolidWorks Corp., Waltham, MA, USA) to design parts and live-linked to COMSOL^®^ Multiphysics (Version 5.3a, Stockholm, Sweden) for FEM analyses. We first used synchronized COMSOL^®^ FEM analysis to study the proposed manipulation system. We then targeted the dimensions of our samples/application and compared the simulation results with the experimental calibration and sample mechanical characterization to observe the operational range and confirm our fabrication conditions.

**Figure 1:**
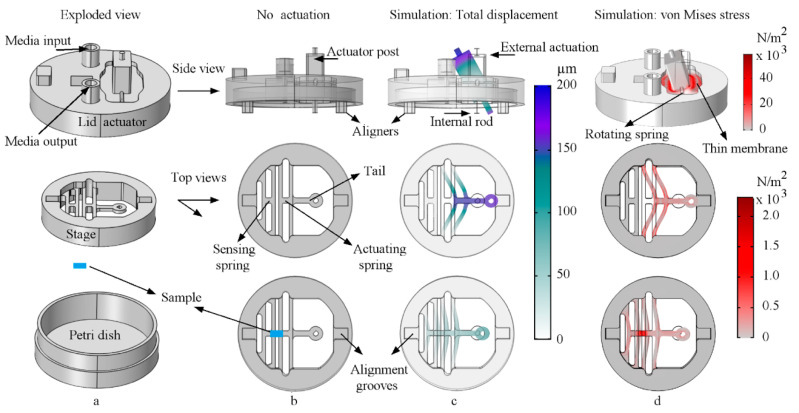
An exploded isometric view of the proposed (**a**, top) lid actuator (media input/output ports allow medium exchange through the lid sterile luer lock blunt needles, syringes, and tubes), (**a**, middle) stage actuator/sensor, and (**a**, bottom) a conventional 35 mm Petri dish. (**b**, top) A side view of the non-stimulated lid actuator, (**b**, middle) top view of the stage without a sample, and (**b**, bottom) the stage with a sample attached (in blue, the samples were fixed to the bottom side, but only the sample on the top side is shown for demonstration). Column (**c**,**d**) show the COMSOL^®^ simulation for total displacement in µm and the von Mises stress in N/m², respectively.

The proposed system includes three main parts: (i) A PDMS lid actuator that transfers external actuation to an internal form, without opening the conventional sterile Petri dish (Figure 1a–d, top); (ii) a PDMS stage, which consists of actuating and sensing springs within a circular shape that fits securely in a 35 mm Petri dish (Figure 1a–d, middle); and (iii) a test sample (PDMS glued, Section 2.6) between the stage springs for mechanical manipulation and characterization (Figure 1b–d, bottom).

The PDMS lid actuator is attached to an external linear actuation arm that pushes the actuator post externally (Figure 1b,c, top). When actuated, the lid rotation spring and thin PDMS membrane surrounding the actuator post deform, allowing a rotation of the lid actuator post (Figure 1c, top). The inner side of the rotating post has a flexible internal rod that also rotates with the post (Figure 1c, top).

The stage actuation spring is pulled by the tail/rod link and transfers actuation into the petri dish (Figure 1b,c, middle). The sensing spring on the other side of the stage (Figure 1b, middle) measures the pulling force delivered through the attached sample. During actuation, the attached sample undergoes tensile stretching and consequently pulls the sensing spring (Figure 1b–d, bottom).

**Figure 2:**
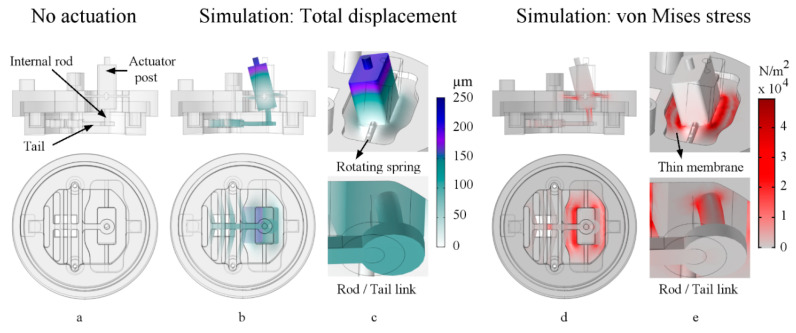
Simulated actuation of the proposed system. The top panels show side views, and the bottom panels show bottom views. Column (**a**) shows the assembly without actuation. Column (**b**,**c**) show the simulated total displacement (µm) under actuation, where (**c**, top) is a top isometric view of the actuator post, and (**c**, bottom) is a bottom isometric view of the rod, and tail link. Column (**d**,**e**) show the simulated von Mises stress (N/m²), where (**e**, top) is a top isometric view of the actuator post, and (**e**, bottom) is a bottom isometric view of the rod and tail link.

Figure 2 shows the details of the actuation mechanism. When the lid, stage, sample, and Petri dish are assembled, the internal rod is linked to the stage’s actuation spring via the tail in a plug and play manner, as shown in the bottom panel of Figure 2c,e.

When assembled, the tail is connected to the internal rod, so when the rod rotates with the post, the rod inversely bends, pulling the tail (Figure 2b–e). Appropriate tuning of the rod/tail link is crucial for two reasons. First, it minimizes the rotational motion component delivered to the actuation spring (via rod bending and tail tilting). Second, it maximizes the linear motion component delivered to the actuation spring (Section 2.5). As a result, when a sample is attached and the actuator post is triggered, the actuation spring stretches the attached sample in an in-plane motion. Subsequently, the sample pulls the sensing spring by the same force. In this manner, not only does the proposed system transfer and deliver controlled mechanical stimuli to a cultured sample as a bioreactor, but it can also characterize the sample’s tensile mechanical properties. Lastly, the assembled system’s Eigen frequencies were simulated, and the lowest frequency was 108.57 Hz, observed for the desired lid rotational motion, as shown in Figure 2b–e, top. Supplementary video (S1) shows the device mechanism from the side, bottom, and top views.

### 2.2. Theory of Operation

**Figure 3:**
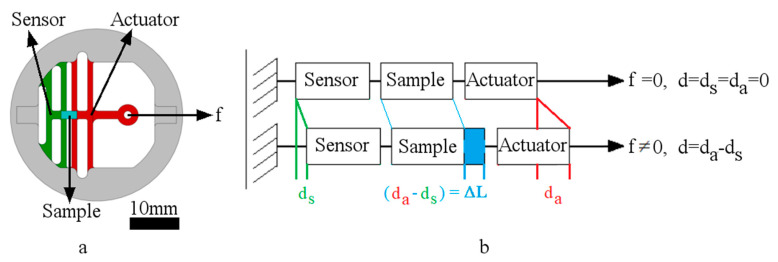
(**a**) Colored stage parts representing (**b**) a schematic diagram of the stage theory of operation (non-actuated vs. actuated) (the actuator is shown in red, the sample in blue, and the sensor in green). Scale bar = 10 mm.

The operation of the stage and attached sample follows Hook’s law for springs in series (within the material’s elastic region and while the sample stress distribution is uniform):

f = k_s_ d_s_ = k ΔL = k_a_ d_a_, (1)

where (f) is the force, (k_s)_ is the constant of the sensor (i.e., the sensing spring), and (d_s)_ is its displacement. (k) is the sample stiffness or spring constant and (ΔL) is its elongation. Finally, (k_a)_ is the spring constant of the actuator (i.e., actuating spring) and (d_a_) is its displacement. Since the sample is attached between the actuating and sensing springs, when tensile stimulation is applied, the resultant sample elongation is equal to the springs’ displacement difference (illustrated in Figure 3):

ΔL = d_a_ − d_s_. (2)

By optically measuring the resultant sample elongation from Equation (2), and the initial actuated length of the sample (L), we can compute the applied strain (ε):

ε = ΔL / L. (3)

By substituting Equation (2) into Equation (1), we can obtain

f = k_s_ d_s_ = k (d_a_ − d_s_) = k_a_ d_a_. (4)

The sensing spring constant (k_s_) is found by the experimental calibration (f = k_s_ d_s_), which we will describe in Section 2.4, while (d_a_) and (d_s_) are optically measured. By plugging these values into Equation (4), we can calculate the sample stiffness (k). Furthermore, by knowing the sample cross-section area (A), we can compute the stress applied to the sample (σ):

σ = f / A. (5)

A critical factor in maintaining the measurement accuracy and theory validity is sustaining a uniform stress distribution through the sample. Actuation thresholds should be established, where a stress distribution uniformity through the sample during actuation is maintained (Section 2.5.3).

By dividing the stress (σ) from Equation (5) over the strain (ε) from Equation (3), we can measure and analyze the sample’s elastic modulus (E):

E = σ / ε.(6)

### 2.3. Fabrication and Materials

As PDMS is the primary material employed in our platform, we mixed a PDMS Sylgard 184 Silicone elastomer (Dow Corning Corp., Midland, MI, USA) base and curing agent at a 10:1 ratio, respectively, throughout this study. An increased hardness/stiffness can benefit some parts of the assembly (e.g., the lid or stiff sensor). However, we avoided the fabrication of PDMS parts using an excessive crosslinking curing agent for a couple of reasons. First, the elasticity is directly affected by the amount of crosslinking agent, and maintaining elasticity supports applications where material bending is involved [28]. Second, PDMS fabrication using the manufacturer protocol (10:1) mixing ratio is highly elastic rather than viscoelastic [29]. Third, flexible PDMS with excess base polymer is preferred for movable structures to obtain actuation without hysteresis [30]. Finally, this approach was adopted to avoid the potential of excess crosslinking agent leaching out into cell culture [31].

#### 2.3.1. Fabrication of Devices

**Figure 4:**
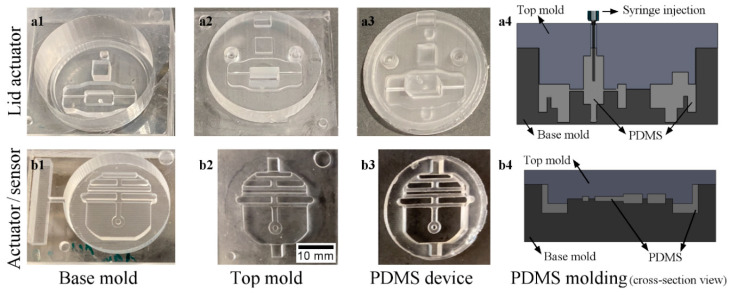
Fabrication (row-**a**) of the lid actuator and (row-**b**) the stage actuator and sensor. (**a1**,**b1**) Acrylic base molds, (**a2**,**b2**) top molds, (**a3**,**b3**) polydimethylsiloxane (PDMS) cured parts, and (**a4**,**b4**) cross-section views of PDMS molding. Scale bar = 10 mm.

The fabrication of the actuator and sensor relied on the molding technique, where we used a CNC milling machine (monoFab SRM-20, Roland DGA corp., Irvine, CA, USA) to micromachine acrylic blocks (McMaster-Carr, Elmhurst, IL, USA) into the top and base master molds. These molds allowed PDMS casting, injection, and degassing before curing. We poured the PDMS mixture into the base mold (Figure 4(a1,b1)) and degassed it for 15 min in a desiccator (Bel-Art Products, Inc., Wayne, NJ, USA). Then, we aligned and pressed the top mold (Figure 4(a2,b2)) to the base mold using vices, sandwiching the elastomer mixture between the molds. The top molds had small openings that allowed PDMS injection and bubble removal using a syringe and blunt needle, as shown in the molds’ cross-sections (Figure 4(a4,b4)).

For comparison and operation range/assembly combination testing, we fabricated the PDMS parts (Figure 4(a3,b3)) in two fabrication/sterilization conditions, as follows:

- Condition 1: Cured PDMS in an oven at 65 °C for 12 h, and then gently peeled it away from the mold and sterilized it using a dry autoclave cycle for 30 min at 121°C (Getinge, Wayne, NJ, USA).
- Condition 2: Cured PDMS in an oven at 65 °C for 2 h, and then gently peeled it away from the mold and disinfected it using isopropyl alcohol (IPA) 70% solution (CiDehol 70, Decon Laboratories, Inc., King of Prussia, PA, USA) for 20 min, followed by sterile distilled water rinses, and left it to dry inside a biosafety hood.

If an autoclave is not available or not a preference, IPA or ethanol 70% can be used for sterilization/disinfection [16,32], and the PDMS stiffness can easily be raised by increasing the curing time [33] or temperature, which has a direct relationship with the cured PDMS hardness and tensile elastic modulus and an inverse relationship with the compressive modulus [34]. Although some studies have argued that, at low temperatures, the PDMS mechanical properties are independent of the curing time, and when increasing the curing temperatures, the PDMS Young’s modulus dropped [35]; their controversial results show curing temperatures of 100–300 °C, not low temperatures. Contrary to the latter study, our observations coincide with an increasing time and temperature increased PDMS stiffness. The PDMS measurements’ hysteresis was reported to relate to manufacturing and testing conditions, while lower curing temperature and cyclic frequency had noticeably reduced hysteresis [36]. Moreover, others have reported on the impacts of the strain rate and the stress–strain curve on the mechanical behavior of PDMS as it relates to its biological application [37]. These studies give a perspective on the fabrication and testing conditions appropriate for each PDMS micromechanical system based on its function/application.

#### 2.3.2. Fabrication of Samples

The surface-treatability and transparency of PDMS facilitate easy cell culture observation experiments. More importantly, the stretchability of PDMS allows for cellular reorientation as a response to delivered cyclic stretching [14], which further provides a biological application previously investigated with established thresholds as a model for the validation of a cellular level bioreactor’s performance.

For the PDMS sheet spin-coating process, the glass side was vacuum-chucked to the spin coater (Laurell technologies corp., North Wales, PA, USA), and the following spinning parameters were used: first cycle: 500 rpm for 10 s, acceleration 100 rpm/s; second cycle: 1400 rpm for 1 min, acceleration 300 rpm/s. Afterward, we placed the glass slide on a hot plate (121 °C) to cure PDMS for one hour. Then, the PDMS sheets were peeled and fixed into a cutting machine (Silver bullet professional, Silver Bullet Cutters ™ LLC., Apple Valley, MN, USA) to cut the PDMS layers into samples with preset dimensions: Length 3 mm × width 1.5 mm, with a gauge length of ~1 mm and thickness of ~30 µm.

On the other hand, the reason why we chose a biodegradable material and induced faster degradation with IPA was to demonstrate many aspects and capabilities at once, as electrospun PLGA nanofiber scaffolds are frequently used in the biomedical regenerative engineering field and have been approved by the FDA for human use [38]. Furthermore, it has been reported that the Young’s modulus of PLGA 85:15 dropped with ethanol treatment [39,40], specifically in a wet state at 37 °C, as opposed to in a dry state at room temperature [41]. Moreover, ethanol (70%) sterilization/disinfection has been reported to result in significant changes to the PLGA scaffolds’ mechanical properties and induce degradation [32]. Another factor that speeds up the scaffolds’ degradation rate is loading [42]. Others have utilized the method in tuning the properties of electrospun polylactide mats by ethanol treatment [43]. These studies suggest an excellent testing specimen for demonstrating the capabilities of the bioreactor.

For PLGA nanofiber (film/scaffold) electrospinning, the fabrication parameters used were 85:15 poly (lactide-co-glycolide) (PLGA) (MW ~94 kDa) (Absorbable Polymers, Birmingham, AL, USA), and Hexafluoroisopropanol (HFIP) from Acros Organics, USA. PLGA (85:15) granules were dissolved at 16% *wt* in HFIP with stirring to form a clear, homogeneous, and viscous solution. PLGA solution was loaded into a 10 mL plastic syringe and electrospun at a flow rate of 0.75 mL/h through a 20-gauge blunt needle with an electrospinning pump (NE 300 SYRINGE pumps, USA) at an applied voltage of 10 kV. The tip of the needle was placed 10 cm away from the collector. The electrospinning process lasted for 3.5 h. Non-aligned electrospun nanofibers were peeled away from the collector and stored in a desiccator until use (>6 months old); films were cut with similar dimensions of length 3 mm × width 1.5 mm, with a gauge length of ~1 mm and thickness of ~91 µm.

A CMOS digital microscopic camera (Chameleon3 CM3-U3-50SM, FLIR Systems Inc., Wilsonville, OR, USA) with a 4−10× objective lens (United Scope LLC dba AmScope, Irvine, CA, USA) was used to monitor the experiments and image the samples’ dimensions. The gauge length (L) and width (W) were then measured using ImageJ (Version 1.52a, National Institute of Health, Bethesda, MD, USA), while the thickness was measured using a digital micrometer (Mitutoyo corp., Takatsu, Kawasaki, Kanagawa, Japan).

### 2.4. Sensor Spring Calibration

The sensing spring calibration took place inside an incubator at 37 °C, 5% CO₂, and controlled humidity. This testing was conducted without the lid actuator or actuation spring.

**Figure 5:**
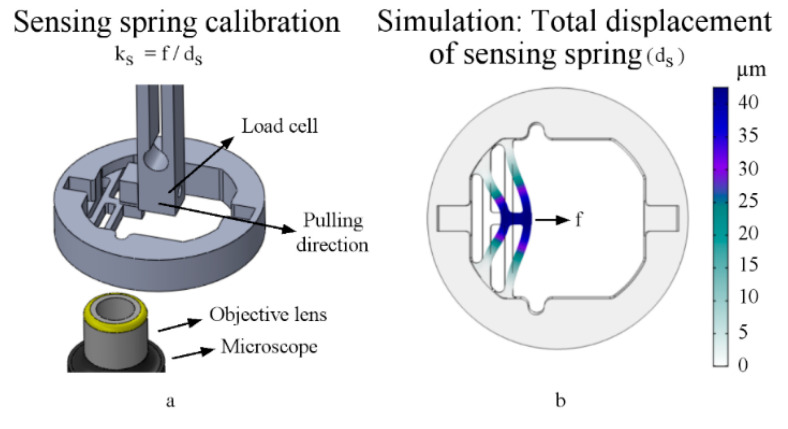
Sensor calibration, where (**a**) is an illustration of the measurement and (**b**) is the matching simulation.

We attached a calibrated load cell to the sensing spring, where the load cell pulled the spring and measured the pulling forces (f). Simultaneously, the microscopic monitoring imaged the consequent sensor displacements (d_s_) (Figure 5a). By utilizing Hook’s law (f = k_s_ d_s_) from Equation (1), we calibrated the stage sensing spring and measured its spring constant (k_s_). On the other hand, we simulated the sensor (Figure 5b) by tuning the PDMS material’s Young’s modulus in COMSOL^®^ until the simulated sensor stiffness (k_s_) best matched the calibrated one; the stiffness matching method reveals the sensitivity of the sensor in each fabrication condition, as shown in the results (Section 3.3).

### 2.5. Operational Range (Computational Tuning Estimations)

#### 2.5.1. Assembly Combinations

**Figure 6:**
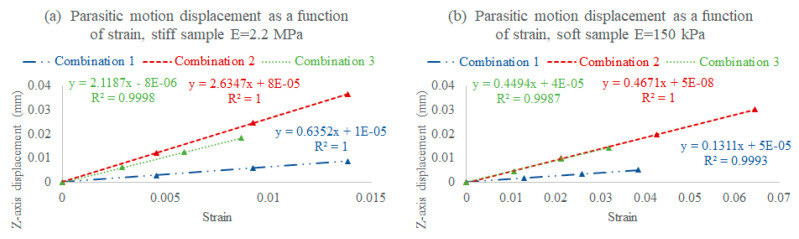
Simulated assembly combination examples showing the strain vs. parasitic displacement relationships (at the sample level) for (**a**) a stiff sample with an elastic modulus of E = 2.2 MPa, and (**b**) a soft sample with an elastic modulus of E = 150 kPa (simulated at fixed sample dimensions and forces). Legends: Combination 1, a stiff lid (3 MPa) and a stiff stage (3 MPa); Combination 2, a soft lid (850 kPa) and a soft stage (850 kPa); and Combination 3, a stiff lid (3 MPa) and a soft stage (850 kPa) (the assembly combinations with the least sample parasitic motion and highest actuator strain and sensor sensitivity for the targeted sample stiffness were chosen for testing).

To estimate and optimize the device operational range and performance, three assembly combinations of two fabrication conditions were simulated on stiff and soft targeted sample moduli; the computational analysis of the assembly combinations, fabrication conditions, and target sample moduli aided in establishing relationships between the strain and consequent parasitic motion displacement at the sample level (Figure 6). These relationships facilitated an appropriate operational range to be employed for each targeted sample, where the optimal scenario for accurate measurements is a high strain and an adequate sensor sensitivity, with the least parasitic motion. The assembly combinations shown below are between fabrication condition 1 (3 MPa) and 2 (850 kPa):

I. Combination 1: A stiff lid (3 MPa) and a stiff stage (3 MPa), where the rod is stiffer, and therefore bends within an acceptable range, increasing the deliverable strain with less parasitic motion, but consequently decreasing the sensor sensitivity (stiffer sensor);
II. Combination 2: A soft lid (850 kPa) and a soft stage (850 kPa), where the lid rod bends too much, increasing the parasitic motion and limiting the deliverable strain while increasing the sensor sensitivity (softer sensor);
III. Combination 3: A stiff lid (3 MPa) and a soft stage (850 kPa), where the rod bends within an acceptable range, slightly reducing the deliverable strain, but also lowering the parasitic motion while maintaining the targeted sensor sensitivity range.

#### 2.5.2. Rod/Tail Link Stiffness Optimization

At the rod/tail link level, the rod stiffness tuning with respect to the targeted sample stiffness is crucial, as it is directly related to how much strain can be delivered to the sample. The strain is limited by the rod/actuation spring linear bending range and sample stiffness. If the rod stiffness is lower than the sample stiffness, more rod-bending takes place than sample-stretching; the opposite is also true, which again stresses the importance of the predictions/simulations for targeted application and early optimization. The rod stiffness optimization was conducted using stiffer samples (PDMS) with known mechanical properties through COMSOL to estimate the operational range and limitations.

The rod/tail link optimization includes increasing the actuation/strain on the *X*-axis while reducing the tail, springs, and sample parasitic motion on the *Z*-axis. Therefore, for better link optimization, we considered a couple of design estimations/considerations based on the axis of movement.

On the stretching *X*-axis, (k _link X_) is the stiffness of the link, while (k _tail X_) and (k _rod X_) are the stiffnesses of the tail and rod, respectively. Similarly, (d _link X_) is the displacement of the link, while (d _tail X_) and (d _rod X_) are the displacements of the tail and rod, respectively.

~ k _link X_ = k _tail X_ + k _rod X_, (very high k _tail X_ & tunable k _rod X_), (7)

~ d _link X_ = d _tail X_ + d _rod X_, (very low d _tail X_ & tunable d _rod X_). (8)

The displacement of the link on the *X*-axis (d _link X_) is defined by its stiffness (k _link X_), where a very high tail stiffness (which must be much stiffer than all other factors, i.e., k _tail X_ >> k_a_, k, k_s_) does not make the tail stretch, but rather translate, based on the rod translation. Therefore, the link stiffness and consequently, its displacements are governed by the rod stiffness (k _rod X_) and its displacement (d _rod X_), which are tuned based on the targeted sample stiffness and the desired strain (within the linear rod displacement range).

Reversely, on the *Z*-axis,

~ k _link Z_ = k _tail Z_ + k _rod Z_, (tunable k _tail Z_ & high k _rod Z_), (9)

~ d _link Z_ = d _tail Z_ + d _rod Z_, (tunable d _tail Z_ & low d _rod Z_). (10)

On the *Z*-axis, the high rod stiffness (diameter or modulus tuned) will result in low undesirable Z-axis movements (low d _rod Z_). The tail stiffness on the z-axis (k _tail Z_) was reduced, by tuning the tail thickness, in order to decrease the link’s parasitic motion, by allowing the tail to slightly tilt, with the rod absorbing most of the rod rotational motion while passing the rod *x*-axis displacement to the actuation spring.

#### 2.5.3. Sample Stress Distribution

**Figure 7:**
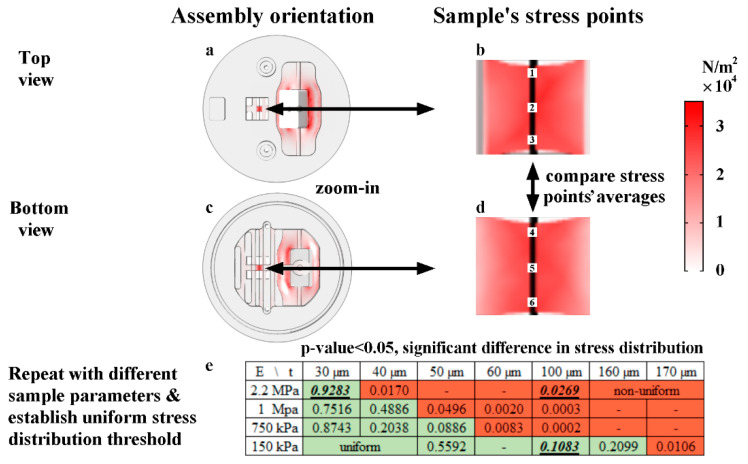
The uniform stress distribution threshold across samples, based on their elastic moduli (E) and thicknesses (t), (*n* = 1 for each simulation thickness and modulus); (**a**) is a top view of the computational analysis (COMSOL); (**b**) is a zoomed-in snapshot of the sample’s top side stress points; (**c**) is a bottom view of the simulation; (**d**) is a snapshot of the sample’s bottom side stress points; (**e**) is the uniform stress distribution threshold table, and the underlined values were tested. PDMS samples had ~2.2 MPa moduli and ~30 µm thicknesses, PLGA films in media had ~2 MPa moduli and ~91 µm thicknesses, and PLGA films in isopropyl alcohol (IPA) 70% had ~10–150 kPa moduli and ~91 µm thicknesses.

The device theory of operation requires the sample stress distribution to be uniform for accurate performances and measurements. Since the samples were attached to the bottom of the stage springs for improved cellular visibility and microscopic tracking, a uniform stress distribution across the sample had to be maintained. Therefore, the stress distribution across stretched samples in COMSOL was simulated (at a fixed sample width, length, and force). At the same time, we altered the sample thickness and elastic moduli to evaluate the stress distribution uniformity for 3D samples with varying elastic moduli. We statistically analyzed three stress points from each side of the thin samples, where samples with no significant difference in the stress distribution from the top to the bottom of the sample were accepted (a uniform stress distribution). In contrast, samples with significant differences between the top and bottom stress points were rejected (a non-uniform stress distribution). A uniform stress distribution threshold based on the sample thickness and elastic moduli was established (Figure 7e) and tested (Section 3.5.2).

The simulation method of samples with varying thicknesses and elastic moduli is shown in (Figure 7); the stress distribution difference between three stress points at the top of the sample vs. three stress points at the bottom of the sample was statistically analyzed and is represented by *p*-values in Figure 7e. If a significant difference in stress averages between the sample sides was found (*p*-value < 0.05), the sample had a non-uniform stress distribution and was therefore out of the operation range (Figure 7e, in red). If no significant difference in the sample stress distribution was found (*p*-values > 0.05), the sample had a uniform stress distribution across the sample and within the operation range (Figure 7e, in green). Three sample groups were tested (underlined in Figure 7e), and the results are discussed in Section 3.5.2.

### 2.6. Surface Treatment, Cleaning, and Sample Attachment Protocols

**Figure 8:**
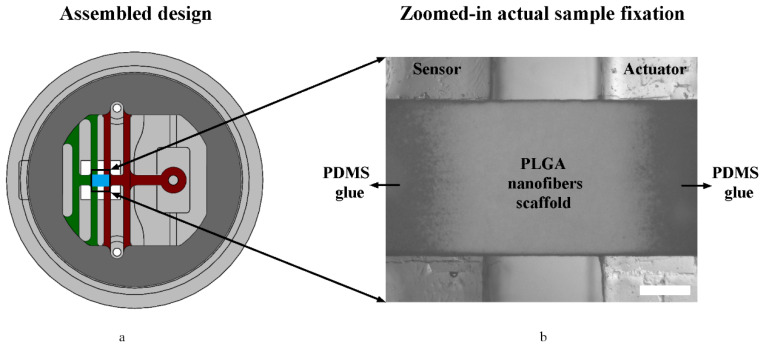
Sample attachment to springs using PDMS as glue: (**a**) shows the assembled design, where the sample is shown in blue, while (**b**) is a zoomed-in image of an actual PDMS-glued specimen (electrospun PLGA 85:15 nanofiber film > 6-months-old). Scale bar = 50 µm.

The PDMS lid and stage surfaces were passivated by immersing them in a pluronic F-127 (P2443, Sigma-Aldrich, St. Louis, MO, USA) solution 0.2% (*w*/*v*) for one hour to block protein attachment [44]. The stage was first placed back into the mold to restrict springs’ movements, in order to attach samples to the springs. A tiny amount of uncured PDMS (10:1) was used as a glue to attach the samples to the stage using the tips of a tweezer; PDMS and PLGA specimens were attached to the treated stage springs from the bottom side and left at room temperature for two-three days to cure, and an example is shown in Figure 8. This method may damage soft samples when peeled as it sustains higher stresses (200–345 kPa) [45]. We gently washed the PDMS parts and samples with sterile distilled water (gibco, via Thermo Fischer, Waltham, MA, USA). Then, we lightly scrubbed any visible dust using cotton swabs and sonicated them in sterile distilled water baths for further cleansing, when needed. Next, we disinfected PDMS parts soaking in IPA 70% baths for 20 min, followed by sterile water rinses and filtered air drying. Following this, we prepared a 20 µg/mL solution of collagen rat tail type 1 (354236, Corning Inc., Corning, NY, USA) in diluted (0.02 N) acetic acid (64-19-7, Sigma-Aldrich). We treated the assembled samples with oxygen plasma cleaner (PDC-32G, Harrick Plasma Inc., Ithaca, NY, USA) for one minute at 18W power (high RF) for surface functionalization [46]. Immediately afterwards, we covered the sample with the collagen solution for one hour at 22 °C to promote protein attachment to the plasma-treated sample, and then gently rinsed it with sterile phosphate-buffered saline PBS pH 7.4 before cell seeding. When storage was required until later use, the untreated PDMS parts were soaked in sterile water in Petri dishes, sealed with parafilm, and kept for up to a week before experiments. We avoided storage for surface-treated PDMS samples, as further attention needs to be taken regarding the surface treatment stability, contact angle, and recovering PDMS hydrophobicity over time [47].

### 2.7. Cell Culture Protocol

We cultured adult Human Dermal Fibroblasts (HDF) (HDF-1N55+, Cascade Biologics, Portland, OR, USA) to confluence (passage 10), in a prepared medium (Dulbecco’s Modified Eagle Medium DMEM, 10% Fetal Bovine Serum FBS, 1% GlutaMAX, 1% Sodium Pyruvate, 1% penicillin-streptomycin (10,000 U/mL)). Cells were rinsed with PBS, suspended using 0.25% trypsin, incubated for three minutes, pelleted by centrifuging at 1200 rpm for 5 min, re-suspended in a fresh medium using a tube shaker for twenty seconds, and seeded on collagen-treated PDMS samples. After cell seeding and four hours of incubation, we gently added more medium until samples were fully submerged. After 20–72 h of cell incubation on PDMS specimens, we maintained a subculture of approximately ~50–100 cell/mm² [16]. Then, we connected the lid actuator to the stage for the dynamic loading and started the experiments on the PDMS specimens for the reorientation and viability evaluations (PBS, DMEM, FBS, GlutaMAX, Sodium Pyruvate, penicillin-streptomycin, and trypsin, gibco, via Thermo Fischer, Waltham, MA, USA).

### 2.8. Mechanical Characterization

The PDMS monolayers were mechanically stimulated in a quasi-static manner, where samples were stretched and the consequent displacements imaged during the brief stops, in order to characterize the sample stiffness using a 0.012/min strain rate and 1.2% strain (end-point).

To demonstrate time-course stiffness measurements at different device operational thresholds and observe the proposed device performance, we applied cyclic quasi-static (0.023 Hz) mechanical stimulation and characterized samples with different ranges of thicknesses and elastic moduli over time.

PDMS monolayer specimens in cell culture media were tested at ~2% strain. Additionally, randomly aligned electrospun PLGA 85:15 nanofibers (>6 months old) were placed in cell culture media at ~2% strain, and to demonstrate the lower stiffness measurement capability, we tested PLGA 85:15 nanofiber films from the same batch and with the same dimensions and thickness, but in IPA 70%, in order to induce rapid degradation at >20% strain; the results are discussed in Section 3.5.2.

The measurements were conducted in a cell culture environment (temperature 37 °C, carbon dioxide CO₂ 5%, and 95–100% humidity, in cell culture media) (except for one group of PLGA 85:15 films was immersed in IPA 70%, *n* = 3). The platform was controlled using customized codes in Matlab^®^ (Version R2019a, Mathworks, Natick, MA, USA) to conduct external actuation, medium perfusion, light illumination, microscopic observation, and image tracking, and aid in sample stiffness characterization.

### 2.9. Cell Stimulation and Viability Testing

#### 2.9.1. Cell Stimulation and Orientation Evaluation

Cell-substrate stimulation to monitor HDF reorientation as a result of PDMS substrate stretching was performed in a dynamic mode (~0.23 Hz) at ~4% strain for 12 h, with 45 s pauses every hour.

For cell reorientation, we measured the angles of cells in four groups (split by polarity into eight groups), consisting of the control at t = 0 and t = 12 h, and stimulated samples at t = 0 and t = 12 h (*n* = 2 for controls, and *n* = 3 for stimulated samples). The angle measuring technique assigns the stretching direction to a zero angle, and cells tilted to the right up to 90 degrees (°) are measured as positive, while cells tilted to the left of the stretching direction are assigned a negative polarity. Cell reorientation before, during, and after 12 h was recorded (Section 3.6.1).

#### 2.9.2. Cell Viability

After culturing in the bioreactor, cells were rinsed with PBS and stained with live/dead assay solution (Invitrogen) at room temperature for 30 min. The viability of cells under a fluorescence microscope was observed (Olympus BX51, Olympus Corporation of the Americas, Center Valley, PA, USA). The viability was monitored after 12 h and one week to show the bioreactor’s biocompatibility with and without mechanical stimulation, respectively (Section 3.6.2).

### 2.10. Statistical Analysis

The results are presented as the mean ± standard deviation or mean ± standard error of the mean (SEM). The type, number of experiments, number of samples, and number of datapoints conducted and evaluated are included in each figure legend or caption. ANOVA single factor, in excel and SASS, was utilized to analyze the results. *p*-values of less than 0.05 (*p*-value < 0.05) were considered statistically significant, and assigned *, and *p*-values < 0.001 were assigned ***.

## 3. Results and Discussion

### 3.1. Assembled System (Design Vs. Actual)

**Figure 9:**
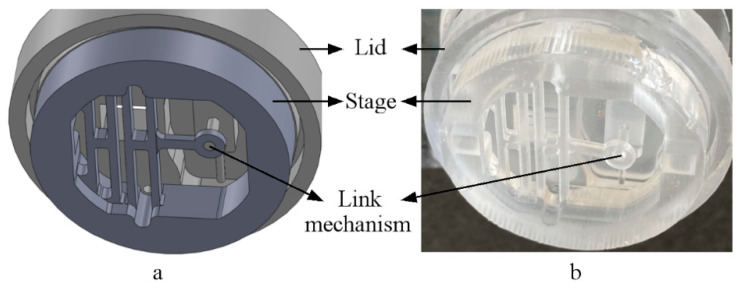
A bottom isometric view of the uniaxial assembled system: (**a**) SolidWorks design and (**b**) actual cured PDMS, showing the tail/rod link mechanism (no sample attached).

The PDMS lid and stage assembly are connected through aligners and a rod/tail link, as previously shown in Figure 1 and Figure 2, respectively. The uniaxial design is compared to the actual assembly in Figure 9. When assembled and a sample is attached, the proposed system offers nutrient exchange, mechanical loading, sensing, and simultaneous monitoring, which does not require opening the dish or manual handling. The assembly mechanism is shown in Supplementary Video (S1).

### 3.2. Assembled System Performance

The platform was placed on top of an inverted microscope (using 4-10x objective lenses), and a cyclic linear motion was applied to the actuator post by an external stepper motor (NEMA 8, Nanotec Plug & drive, Nanotec Electronic GmbH & Co KG, Stoneham, MA, USA). At the same time, image tracking of the springs/sample and displacements/deformations, respectively, was conducted using customized codes in Matlab^®^ (Version R2019a, Mathworks Inc., Natick, MA, USA).

#### 3.2.1. On the *X*-Axis (Repeatability)

The bioreactor’s repeatability was very compliant to the external actuation motor accuracy. Thorough testing of the stepper motor was conducted, with and without PDMS parts. A ±5% non-accumulative error was present in all cases due to the stepper motor cyclic backlash, as reported by the manufacturer [48] and others [49,50].

In total, seventeen thousand two hundred and eighty images and 8640 strain peak data points were analyzed for each PDMS specimen/stage (*n* = 3), with HDF cells cultured and sensors being engaged for a dynamic repeatability evaluation, where the motor was set to deliver 4% strain in a high/low mode at 0.23 Hz. In all three samples/stages, the repeatability tests had standard deviations of ±0.2% strain anticipated for the stepper motor cyclic backlash error (±5%).

**Figure 10:**
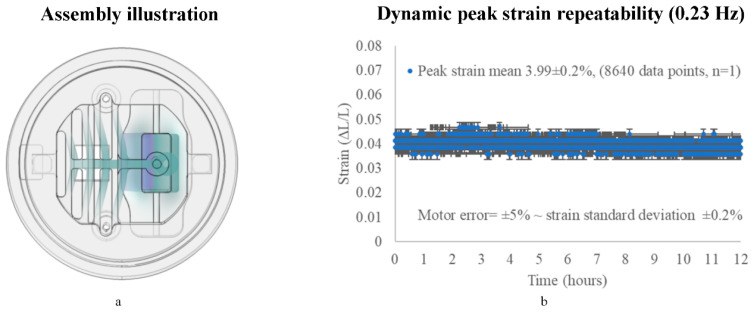
PDMS assembly stretching mechanism with a PDMS monolayer attached and sensor engaged, where (**a**) is a simulation/illustration of the stretching mechanism and (**b**) is the dynamic strain repeatability for 12 h at 0.23 Hz (8640 high peak data points); *n* = 1. Measured data are presented as the mean ± standard deviation (error bars).

To discuss the platform repeatability while excluding factors such as sample variations in dimensions or the installation accuracy, an average strain of one sample/stage (8640 cycles, *n* = 1) is presented in Figure 10, where the average peak strain was 3.99 ± 0.2% for 12 h.

Overall, our findings reinforce the platform performance consistency to the resolution level of tuning, the external motor, the imaging devices, the sample dimensions, and its installation.

#### 3.2.2. On the *Z*-Axis (Parasitic Motion)

In the computational analysis, when actuated, no significant difference was found between the actuation and sensing springs at the sample gluing site, with both having similar slightly increasing levels on the *z*-axis as the strain increased, indicating the possibility of focal length synchronization for high strain/stiff samples if needed.

The tuned bioreactor operated with reduced parasitic motion displacement on the z-axis, which was barely noticeable under the microscope (during *x*-axis measurements). Although the parasitic motion-induced error was measured at higher strains, we managed to keep it within the stepper motor cyclic error range of ±5% standard deviations.

The prediction method applied in Figure 6 regarding the assembly combination, strain, sample moduli, and resultant *z*-axis displacements helped in fine-tuning and reducing the parasitic motion effects to negligible levels (within the motor error). For this reason, we stress the importance of simulation/prediction for obtaining an optimized performance and reducing unforeseen errors.

### 3.3. Sensor Stiffness (k_s_) Calibration (No Sample Attached)

**Figure 11:**
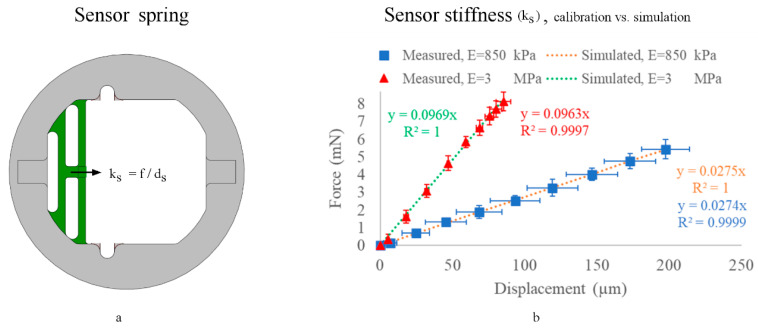
Calibration of sensing spring constants (k_s_). (**a**) is an illustration, while (**b**) is the measured sensing spring constants vs. simulated ones, with *n* = 5 for each fabrication condition. The simulated stiffness equation for fabrication condition 1 (in green) is y = 0.0969x (R² = 1), and the measured value (shown in red) is y = 0.0963 (R² = 0.9997). The simulated stiffness equation for fabrication condition 2 (in orange) is y = 0.0275x (R² = 1), and the measured value (shown in blue) is y = 0.0274x (R² = 0.9999). Measured data are presented as the mean ± standard deviation (error bars).

The sensor calibration and stiffness matching show the spring sensitivity displacement as a linear function of applied force in certain fabrication conditions (Figure 11). The measured elastic moduli for fabrication (conditions 1 and 2) were E = 3 MPa and 850 kPa, respectively.

We demonstrated the measured vs. simulated sensing spring constants (k_s_) for each fabrication condition, presenting the fabrication (curing/tuning) condition sensitivity. The measured mechanical properties of molded PDMS varied, depending on the fabrication conditions, which confirms that the elasticity of cured molded PDMS is time- and temperature-dependent [34,51], and shows how the operation, manipulation, and characterization range is highly tunable, based on the PDMS curing conditions [33,34].

### 3.4. Actuator Stiffness (k_a_) Characterization (with a Sample Attached)

**Figure 12:**
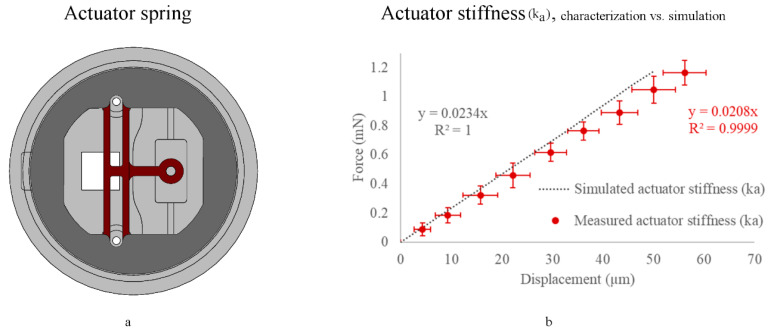
Characterization of the actuator spring constant (k_a_) (while a sample is attached), where (**a**) is an illustration, while (**b**) is the measured actuating spring constant (k_a_) vs. its simulation (*n* = 9, stage fabrication condition 2). The simulated stiffness equation for the actuation spring (shown in red) is y = 0.0234x (R² = 1), and the measured stiffness (shown in gray) is y = 0.0208x (R² = 0.9999), where measured data are presented as the mean ± standard deviation (error bars).

When an elastic sample (PDMS) is attached to the platform, the actuator, sample, and sensor act as three springs in series; once external stimulation is applied, the actuator is displaced by (da), elongating the sample by (ΔL), and consequently, the sample displaces the sensor by (ds) (illustrated in Figure 3). Therefore, we measured the actuator (da) and sensing (ds) spring displacements to calculate the sample elongation (ΔL) from Equation (2). Furthermore, to obtain the applied force (f) and the stiffness of the actuator spring (ka), we substituted the measured displacements (ds), (ΔL), and (da), along with the calibrated sensor stiffness (ks), into Equation 4. The resultant actuator stiffness (ka) versus its simulation is shown in Figure 12b.

Through the actuator characterization method, we found the actuator (spring/rod) performance to be linear within the (spring/rod) linear bending limits (which is highly tunable, based on the targeted application, curing conditions, and design).

### 3.5. Sample Mechanical Characterization

From the same substitution in Equation (4), the measured sample stiffness (k) was obtained. However, since the sample stiffness (k) is the geometric property, while Young’s modulus (E) is the material property, we present the sample mechanical properties through the elastic modulus (E).

#### 3.5.1. End-Point

After measuring the sample stiffness (k), sample length (L), and its elongation (ΔL), we computed the sample’s strain (ε) from Equation (3), stress (σ) from Equation (5), and Young’s modulus (E) from Equation (6). 

**Figure 13:**
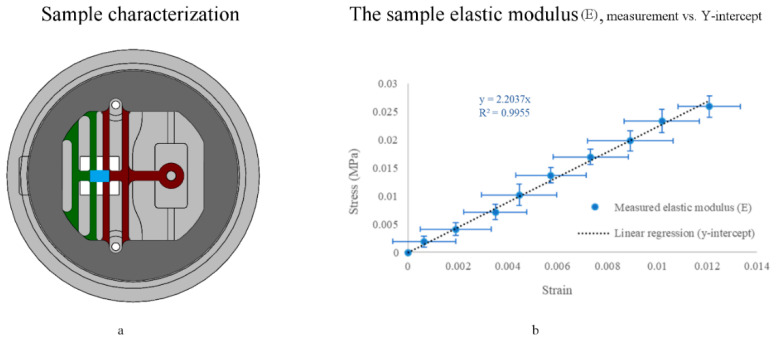
PDMS specimen Young’s modulus E characterization. (**a**) is an illustration (sample shown in blue), and (**b**) is an end-point measurement of a spin-coated PDMS sample (E = 2.2 MPa) (blue, *n* = 9) vs. linear regression (in black). The elastic modulus equation for the linear regression is y = 2.2037x, R² = 0.9955. Measured data are presented as the mean ± standard deviation (error bars).

The elastic modulus was quasi-statically measured, averaged, and plotted versus its estimated linear regression, as shown in Figure 13. Our findings are comparable to what has been previously reported [34], where PDMS samples (Sylgard 184, prepared at a 10:1 mixing ratio) that were stretched by low strains displayed linear behavior, allowing the calculation of Young’s modulus using Hooke’s law (Equation 6). We measured a 2.2 MPa elastic modulus (*n* = 9) at 1.2% strain.

#### 3.5.2. Time-Course

Time-course mechanical stimulation and stiffness characterization of PDMS sheets and electrospun PLGA 85:15 nanofiber films were conducted. The bioreactor measured an average modulus fluctuating around ~2 MPa at ~2% strain, in cell culture media, while PLGA films in IPA 70% instantly shrunk and degraded, and their modulus dived into the softer moduli range (10–150 kPa) at strains of >20%, where all groups (*n* = 3 each) were under tensile cyclic quasi-static stretching (at 0.023 Hz).

**Figure 14:**
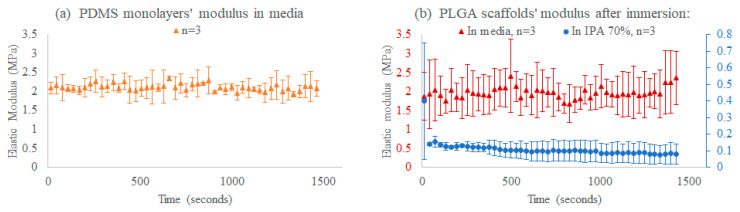
Time-course mechanical characterization of (**a**) stiff PDMS sheets in media (shown in orange, *n* = 3), and (**b**) PLGA 85:15 film in media (shown in red, *n* = 3) and in IPA 70% (shown in blue, *n* = 3). Measured data are presented as the mean ± standard deviation (error bars).

To assess the predictions presented in Section 2.5, we tested samples in, above, and below the device operating range. Figure 14a shows PDMS monolayers in media with moderate elastic modulus fluctuation around the modulus mean. For this group, the PDMS sheets were, in fact, right below the thickness and modulus thresholds (Figure 7e), which may explain the slight variations in measurements.

Furthermore, Figure 14b (in red) shows the elastic modulus measurements of non-aligned PLGA nanofiber films in media, with significant deviations around the mean and non-repeatability in the delivered strain; this group was above both the modulus and thickness thresholds (a non-uniform stress distribution), abiding by our simulations regarding the rod stiffness compared to the sample stiffness (the rod must be stiffer than the sample on the axis of stretch to obtain a linear performance within the rod linear bending range).

From the same PLGA 85:15 film batch, Figure 14b (in blue) shows the instant average moduli drop into the acceptable range right after immersion in IPA 70%, which induced rapid PLGA degradation, softening, and noticeable structural changes. This group instantly dropped in moduli, allowing it to fall into the uniform stress distribution range (below the operation range threshold, shown in Figure 7e); consequently, far fewer measurement deviations were observed.

The three groups’ results, shown in Figure 14, comply with the predicted device’s operation range in Figure 7e. Our findings present the capability of the bioreactor to quantify the elastic moduli of samples in the order of tens to hundreds of kPa overtime.

### 3.6. Cell Reorientation Validation Experiments

To test the bioreactor on the cellular level over an extended time, we followed well-defined literature-based cell reorientation trends and thresholds. To successfully re-orient HDF cells cultured on PDMS membranes, the stretching should be above the lower thresholds of the HDF reorientation frequency (0.1 Hz) and strain (2%). Since it has been reported that 1–5 h is sufficient for cell reorientation to occur based on the applied frequency and strain [16] and that lower strain decreased the degree of perpendicular orientation [14,15], the time needed for reorientation increased [14,16].

On the other hand, frequencies above 1 Hz saturated the characteristic time [16], and prolonged high frequencies, combined with high strain for an extended period, disturbed cells’ adhesion [17]. Similarly, an upper axial strain limit of 4.2 ± 0.4% was reported, and above this value, fibroblasts were not present [15].

Therefore, to prolong the dynamic loading while maintaining cell adhesion, the cells’ lower and higher frequency and strain thresholds with respect to the loading time were considered. Therefore, a frequency of 0.23 Hz and a strain of 4% were chosen to maintain cell adhesion and viability [17], while observing slower cells’ reorientation trends for 12 h [16].

#### 3.6.1. Cells’ Reorientation Trends

**Figure 15:**
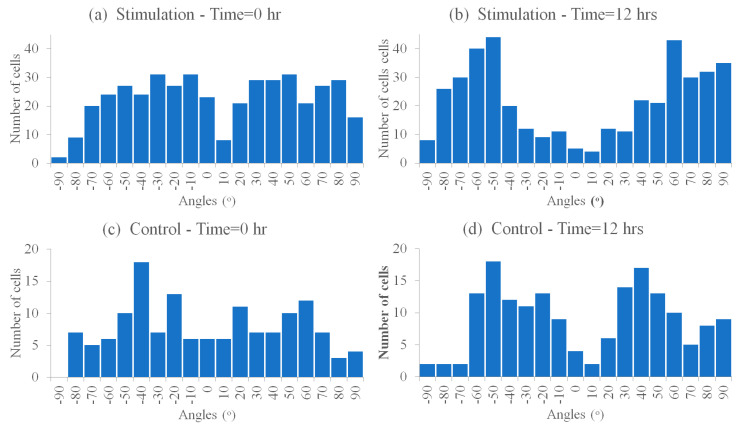
Histograms showing cells’ angle distribution, where (**a**) is the stimulation at t = 0 h, cells# 429; (**b**) is the stimulation at t = 12 h, cells# 415; (**c**) is the control at t = 0 h, cells# 145; and (**d**) is the control at t = 12 h, cells# 170 (cell angle data were pooled from stimulated samples, *n* = 3, and from control samples, *n* = 2).

The HDF cells’ reorientation trends were monitored and analyzed to evaluate the bioreactor’s mechanical stimulation delivery to biological samples. The HDF response to substrate stretching before, during, and after 12 h was observed (*n* = 2 control, *n* = 3 stimulated). The distribution of cells’ reorientation angles is shown in Figure 15, where the trend is noticeable in Figure 15b. 

**Figure 16:**
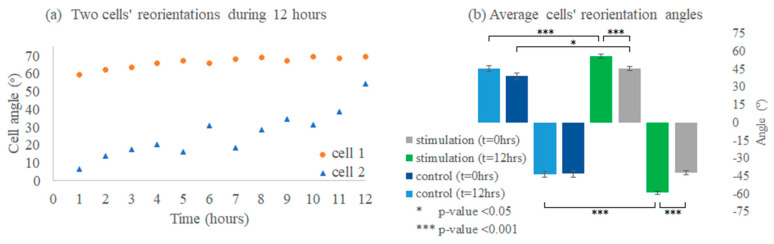
Cells’ reorientation profiles, where (**a**) shows two cells’ reorientations over 12 h (starting from different angles), while (**b**) shows the average cells’ angles before and after 12 h (group analysis of the same angle polarity), where gray bars display stimulation at t = 0 h, where cells# 429 had 212 negative (neg.), and 217 positive (pos.) angled cells; green bars display stimulation at t = 12 h, where cells# 415 had 203 neg. and 212 pos. angled cells; navy bars display the control at t = 0 h, where cells# 145 had 77 neg. and 68 pos. angled cells; and blue bars display the control at t = 12 h, where cells# 170 had 85 neg. and 85 pos. angled cells. Data pooled from stimulated samples, *n* = 3, and for the control, *n* = 2; measured data are presented as the mean ± standard error of the mean (SEM error bars).

**Figure 17:**
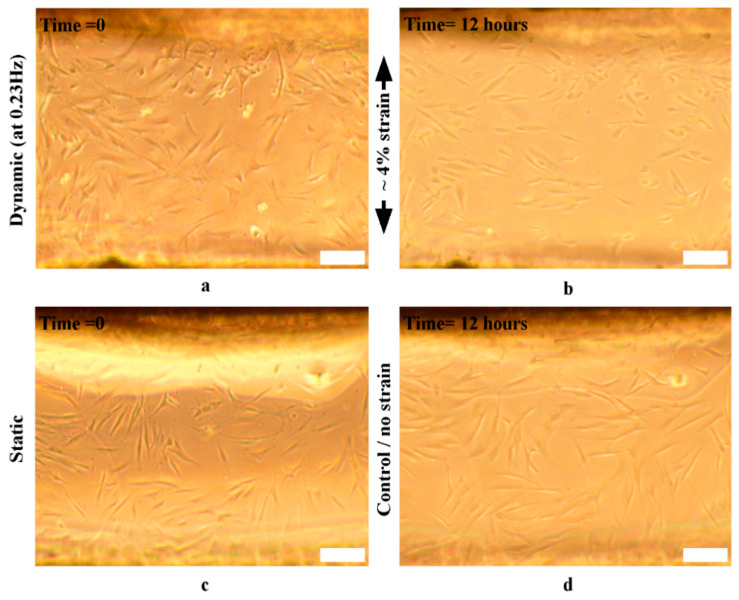
Human dermal fibroblast (HDF) cells cultured on PDMS monolayers mechanically stimulated (*n* = 3) vs. the control (*n* = 2) before and after 12 h, where (**a**) is the stimulated sample at time = 0 h, (**b**) is the stimulated sample at t = 12 h at 0.23 Hz and ~4% strain, (**c**) is the control at t = 0 h, and (**d**) is the control at t = 12 h. Scale bars = 20 µm.

Our results match the literature, where the stimulated cells exhibited a significant difference in cell angles due to dynamic loading, while controls did not display a significant difference (before and after 12 h), as shown in Figure 16b. Furthermore, although cells oriented away from the stretching direction (angle 0°) towards two well-defined angle regimes (averages −58°, and +56.02°), shown in Figure 15b, Figure 16b, and Figure 17b, as reported in [14], cell reorientation certainly took a longer time to occur [17]. Furthermore, the distribution profile of cell reorientation angles in Figure 15b for the two well-defined angles was not as sharp as previously reported under a higher frequency and strain [14].

It was apparent from image tracking and analysis that cell reorientation was gradual; Figure 16a shows two measured cells’ orienting their direction or maintaining it away from the stretching direction, regardless of the initial cell angle, complying with the reported testing trends [14,17]. The slower cell orientation trends observed were due to the applied strain and frequency amplitudes. Therefore, cellular adhesion was also maintained over 12 h, as opposed to previously reported cell disturbance at a higher strain and frequency over a shorter time [17].

Examples of cells’ reorientation before and after 12 h are shown in Figure 17, for all groups. These results demonstrate the ability of the PDMS bioreactor to apply micro-cellular manipulations with a predicted performance based on the application or purpose of the test.

#### 3.6.2. Viability

Finally, to verify the platform’s biocompatibility, we conducted cell viability assays to study the setup cytotoxicity in two conditions: After 12 h of dynamic mechanical stimulation and after one week of static cell culture (no stimulation).

**Figure 18:**
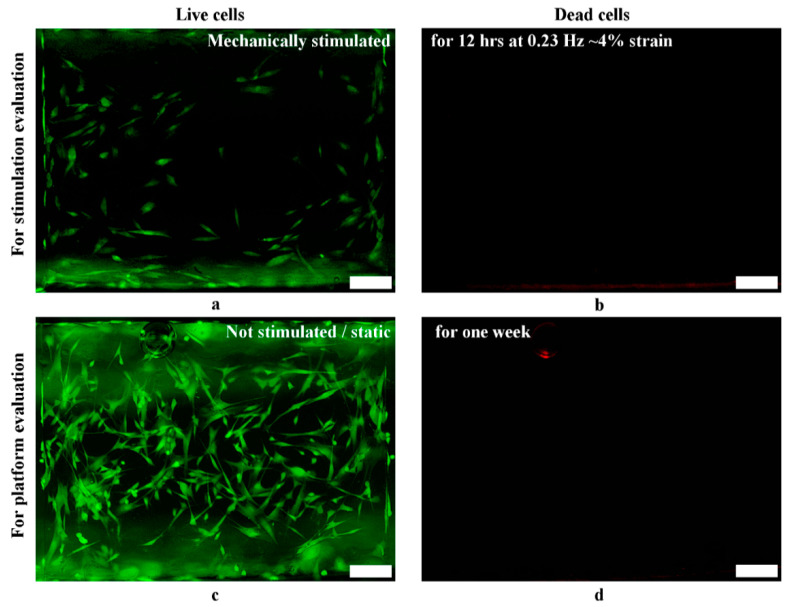
Viability of HDF cultured on PDMS specimens (**a**) after stimulation for 12 h at 0.23 Hz and ~4% strain, showing live cells in green (FITC filter), and (**b**) after stimulation, showing no dead cells in red (Texas red filter). (**c**) presents HDF cells without mechanical stimulation after one week of culturing, showing live cells in green, and (**d**) presents those without mechanical stimulation after one week of culturing, showing no dead cells in red (Figure **c** and **d** had the same air bubble). For stimulation, *n* = 3, and for platform evaluations, *n* = 5. Scale bars = 20 µm.

In both cases, a lack of dead cells or contaminants was maintained (Figure 18), suggesting the biocompatibility of the proposed platform and applied protocols for biological applications.

### 3.7. Future Direction

Biaxial mechanical stimulation has previously been investigated, and its importance has been demonstrated; for instance, human articular chondrocytes were found to respond to both uni- and biaxial mechanical stimulation while encapsulated in hydrogels composed of hyaluronic acid methacrylate (HAMA) and gelatin methacryloyl (GelMA). The mechanical stimuli enhanced the hyaline cartilage-specific (HRS) extracellular matrix accumulation and HRS marker gene upregulation [52]. Others reported that biaxial stretching enhanced cardiomyocytes’ maturation [53].

Our device has four main bioreactor components: (1) An actuator and motion-transformation mechanism to deliver designed mechanical stimuli; (2) a control and monitoring system to precisely apply loading profiles and obtain deformation, respectively; (3) a sterile chamber to provide a tissue/cell culture environment; and (4) nutrition perfusion features to exchange waste with new media [21,22,23]. For obtaining more functionality, a future direction may be a bi-axial tunable PDMS bioreactor with combined bio-tissue micromanipulation and stiffness detection roles.

## 4. Conclusions

The novel PDMS-based bioreactor offers time-course features including, and not limited to, cell culture, media/nutrient perfusion, mechanical stimuli, stiffness characterization, degradation quantification, and live imaging, and can accommodate various targeted samples via fabrication tuning.

The platform repeatedly delivered time-course quasi-static strain of over 20% to (induced) degrading PLGA nanofiber films and measured elastic moduli of 10–150 kPa, while stretched stiff PDMS monolayers exhibited up to ~4% strain and displayed moduli of ~2.2 MPa. The apparatus dynamically (0.23 Hz) applied tensile stimuli to live HDF cells cultured on PDMS sheets and recorded the cellular reorientation trends for 12 h, with no cellular toxicity concerns. Similarly, the viability was maintained for one week without mechanical stimulation.

The platform fully demonstrated several combined capabilities without opening the Petri dish or exposing the sample, which reduced cross-contamination. The low-cost micromechanical apparatus delivers and combines bioreactor functions with predicted/tunable performances. At the same time, it eliminates the need for an unjustified pricy, bulky experimental setup, increasing the ease of live-cell manipulation studies by providing an affordable way to conduct high-throughput experiments. The properties, accessibility, biocompatibility, tunability, and wide-use of PDMS material make the adaptation and custom modification easy. To conclude, the proposed system is advantageous for low-cost cell culture micromanipulation studies, which include both mechanical stimulation and force characterization within user-defined operational ranges.

## Supplementary Materials

The following are available online at www.mdpi.com/xxx/S1: Video S1: The mechanism of the PDMS bioreactor.
